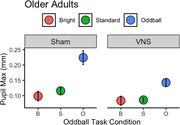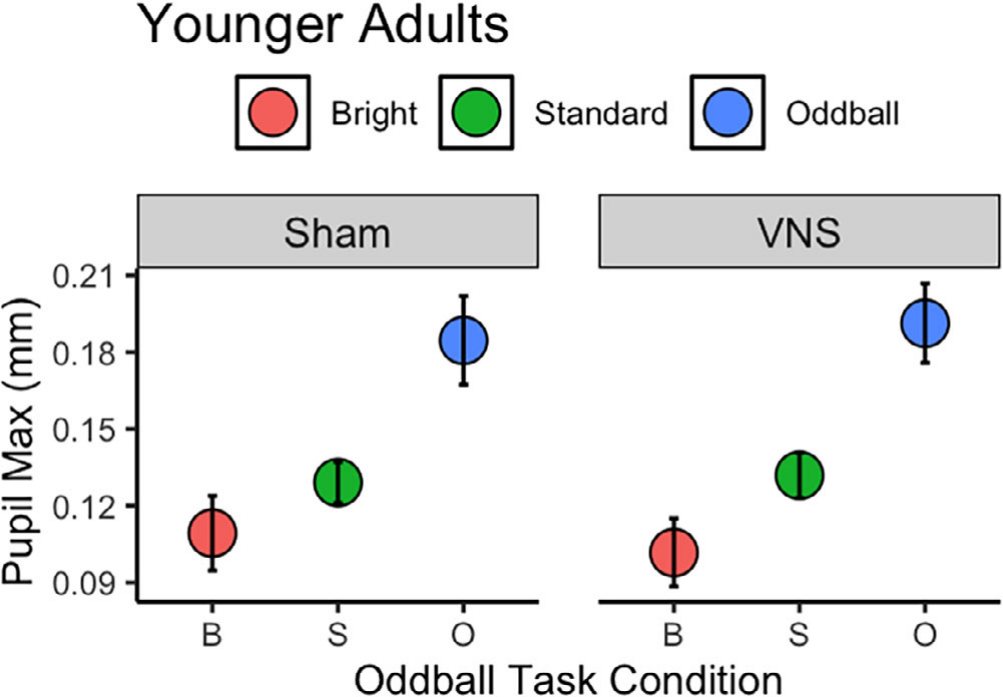# Effects of transcutaneous auricular vagus nerve stimulation on pupillary responses in younger and older adults

**DOI:** 10.1002/alz70857_100274

**Published:** 2025-12-25

**Authors:** Elizabeth Riley, Genevieve Wager, Eve De Rosa, Adam Anderson

**Affiliations:** ^1^ Cornell University, Ithaca, NY, USA

## Abstract

**Background:**

The locus coeruleus (LC) is an early site of neurodegeneration in Alzheimer's disease. Its health and functional status are important correlates of, and potentially mediators of, disease progression. Improving the functional health of the LC may be possible using transcutaneous auricular vagus nerve stimulation (taVNS) to modulate neuronal activity in the region and modify norepinephrine release. However, since the structure and function of the LC change across the lifespan, it is unknown whether the effects of taVNS are similar in younger vs. older adults.

**Method:**

21 participants (66% female, 12 under age 50, 9 over age 50) received both sham (earlobe) and verum (cymba concha) taVNS for 7 minutes each (separated by an average of 38.9, 44.2 SD) while doing a visual oddball task (60% standard stimuli, 20% oddball stimuli, 20% brighter standard stimuli to control for pupillary light reflex) with concurrent pupillometry. taVNS stimulation parameters were adjusted individually for strong sensation, with current ranging from 900 to 3200 μA, 25 Hz frequency, 250 μs pulse width, and 50% duty cycle: 30/30 sec on/off. Pupillary analysis included the calculation of the maximum pupil diameter in mm above pretrial baseline for each trial.

**Result:**

We constructed a linear mixed effects model on maximum pupil diameter for oddball stimuli with fixed effects of treatment (sham vs. verum taVNS) and age group, and a random effect of participant. Using joint tests, we found a significant effect of treatment on oddball responses (F(1,1165.93) = 6.84, *p* =  0.009), and a significant interaction between age group and treatment (F(1,1165.93) = 11.52, *p* =  0.0007). Marginal means demonstrated that in the younger adults, pupillary maxima increased nonsignificantly during verum taVNS compared with sham (3.7% larger, *p* =  0.50), while in older adults, pupillary maxima decreased significantly (30.5% smaller, *p* =  0.0002).

**Conclusion:**

In this pilot study, taVNS appears to have unique effects on pupillary response size to visual oddballs in younger adults vs. older adults, suggesting underlying differences in LC function between age groups.